# Integrated proteomic and N-glycoproteomic analyses of doxorubicin sensitive and resistant ovarian cancer cells reveal glycoprotein alteration in protein abundance and glycosylation

**DOI:** 10.18632/oncotarget.14542

**Published:** 2017-01-06

**Authors:** Yanlong Ji, Shasha Wei, Junjie Hou, Chengqian Zhang, Peng Xue, Jifeng Wang, Xiulan Chen, Xiaojing Guo, Fuquan Yang

**Affiliations:** ^1^ Laboratory of Protein and Peptide Pharmaceuticals and Laboratory of Proteomics, Institute of Biophysics, Chinese Academy of Sciences, Beijing 100101, China; ^2^ University of Chinese Academy of Sciences, Beijing 100049, China; ^3^ National Laboratory of Biomacromolecules, Institute of Biophysics, Chinese Academy of Sciences, Beijing 100101, China

**Keywords:** SILAC, global proteome, N-glycoproteome, multidrug resistance, ovarian cancer

## Abstract

Ovarian cancer is one of the most common cancer among women in the world, and chemotherapy remains the principal treatment for patients. However, drug resistance is a major obstacle to the effective treatment of ovarian cancers and the underlying mechanism is not clear. An increased understanding of the mechanisms that underline the pathogenesis of drug resistance is therefore needed to develop novel therapeutics and diagnostic. Herein, we report the comparative analysis of the doxorubicin sensitive OVCAR8 cells and its doxorubicin-resistant variant NCI/ADR-RES cells using integrated global proteomics and N-glycoproteomics. A total of 1525 unique N-glycosite-containing peptides from 740 N-glycoproteins were identified and quantified, of which 253 N-glycosite-containing peptides showed significant change in the NCI/ADR-RES cells. Meanwhile, stable isotope labeling by amino acids in cell culture (SILAC) based comparative proteomic analysis of the two ovarian cancer cells led to the quantification of 5509 proteins. As about 50% of the identified N-glycoproteins are low-abundance membrane proteins, only 44% of quantified unique N-glycosite-containing peptides had corresponding protein expression ratios. The comparison and calibration of the N-glycoproteome versus the proteome classified 14 change patterns of N-glycosite-containing peptides, including 8 up-regulated N-glycosite-containing peptides with the increased glycosylation sites occupancy, 35 up-regulated N-glycosite-containing peptides with the unchanged glycosylation sites occupancy, 2 down-regulated N-glycosite-containing peptides with the decreased glycosylation sites occupancy, 46 down-regulated N-glycosite-containing peptides with the unchanged glycosylation sites occupancy. Integrated proteomic and N-glycoproteomic analyses provide new insights, which can help to unravel the relationship of N-glycosylation and multidrug resistance (MDR), understand the mechanism of MDR, and discover the new diagnostic and therapeutic targets.

## INTRODUCTION

Ovarian cancer is one of the most commonly diagnosed malignant tumors that threaten the health of women. A total of 22,280 new cases and 14,240 deaths that will occur in the United States in 2016 are estimated by the American Cancer Society [1]. Currently, chemotherapy plays a pivotal role in the treatment of ovarian cancer patients treated with survey. Among chemotherapeutic agents, doxorubicin, also known as adriamycin, which belongs to the anthracycline antibiotic, is one of the most commonly used drugs in the treatment of ovarian cancer and other solid tumors [2]. Cancer cells always develop resistance to doxorubicin after the plentiful and permanent clinical application of this drug in cancer treatment. Tumor cells become resistant not only to the drug used but also to a great variety of structurally and functionally unrelated compounds as long as they develop drug resistance. MDR is a major problem towards the successful treatment of ovarian cancer and the main cause of the failure of chemotherapy [3, 4]. To date, several theories have been proposed to account for the development of MDR such as increased drug efflux and DNA damage repair, reduced drug uptake, altered drug target expression and resistance to apoptosis [5, 6]. However, the underlying mechanisms of MDR in human ovarian cancer are still not completely elucidated.

Glycosylation, one of the most important and complex post-translational modifications in nature [7], plays an important role during molecular and cellular recognition and communication [8], especially in cancer progression [9] and immune responses [10, 11]. One mechanism of MDR is to increase drug efflux by overexpression of ATP-binding cassette transporters, such as multidrug resistant protein (P-glycoprotein/ABCB1) [12, 13]. multidrug resistance-associated protein 1 (MRP1/ABCC1) [14–16], and breast cancer resistance protein (BCRP/ABCG2) [17–19]. All transporters are N-linked glycosylated membrane glycoproteins. Aberrant glycosylation is known to be associated with various human diseases [20]. Recently, a number of studies focused on identifying the correlation between glycosylation of glycoproteins that are expressed specifically in tumor cells and chemoresistance [21–24]. Wojtowicz et al. showed that inhibition of protein N-glycosylation reverses the MDR phenotypes of cancer cell lines [25]. Zou et al. found that 15 proteins included ABCB1 as well as a set of novel glycoproteins such as CTSD, FKBP10 and SLC2A1 associated with chemoresistance in leukemia cells. Fan et al identified and quantified 11 membrane N-glycoproteins which are significantly changed in MDR gastric cancer cells and they are helpful for better interpreting the sophisticated mechanisms of MDR in gastric cancer [22]. Di Michele et al screened the differential expressed glycoproteins between drug sensitive and resistant cell lines and found 4 glycoproteins remarkably upregulated in drug resistant cells as putative biomarkers for paclitaxel resistance in ovarian cancer [24]. These studies have demonstrated the N-glycosylation alterations in the drug resistant cancer cell lines compared to the drug sensitive cancer cell lines, but systematic and comprehensive information is still unavailable on the correlation of N-glycosylation with multidrug resistance.

In this study, Hydrophilic interaction liquid chromatography (HILIC) based enrichment combining with SILAC labeling strategy was established, optimized and used to compare the differences of N-glycosylation level between OVCAR8, a common ovarian cancer cell line and the doxorubicin-resistant cell line NCI-ADR/RES, which is derived from OVCAR8 by continuous *in vitro* exposure to increasing concentrations of doxorubicin. The significantly changed N-glycoproteins and N-glycosites have been found, which might be related to MDR. This will be helpful to understand the mechanisms of MDR.

## RESULTS

### Evaluation of the optimized HILIC based enrichment method for N-linked glycopeptides

Three equal aliquots of 600 μg proteins from mouse brain tissue, NCI/ADR-RES and OVCAR8 cells were digested using trypsin respectively, and used to optimize the HILIC based enrichment method for N-glycopeptides, and evaluate the enrichment efficiency and specificity. In mouse brain, LC-MC/MS analysis identified 1542, 1356 and 1507 high confidence N-glycosites in triplicates. 49.5% of the N-glycosites were identified in all the three replicates, and nearly 74% of the N-glycosites were identified at least twice in the three replicates (Supplementary Table 1, Supplementary Figure 1A), A total of 1957 N-glycosites were identified, covering most of the N-glycosites in mouse brain which were found in the previous study [26]. Furthermore, the enrichment specificity was 86.9%, 87.5% and 84.9% of each single run separately. To be our knowledge, this should be the highest enrichment specificity for HILIC-based enrichment reported so far (Supplementary Table 2). The similar results were also obtained from OVCAR8 and NCI/ADR-RES cells (Supplementary Tables 3 and 4, Supplementary Figure 1B and 1C). All of the above results demonstrated that our methods had a good stability, reproducibility, high enrichment specificity, and that the data set was highly confident.

### N-glycoproteomic analyses of OVCAR8 and NCI/ADR-RES cells

**Figure 1:**
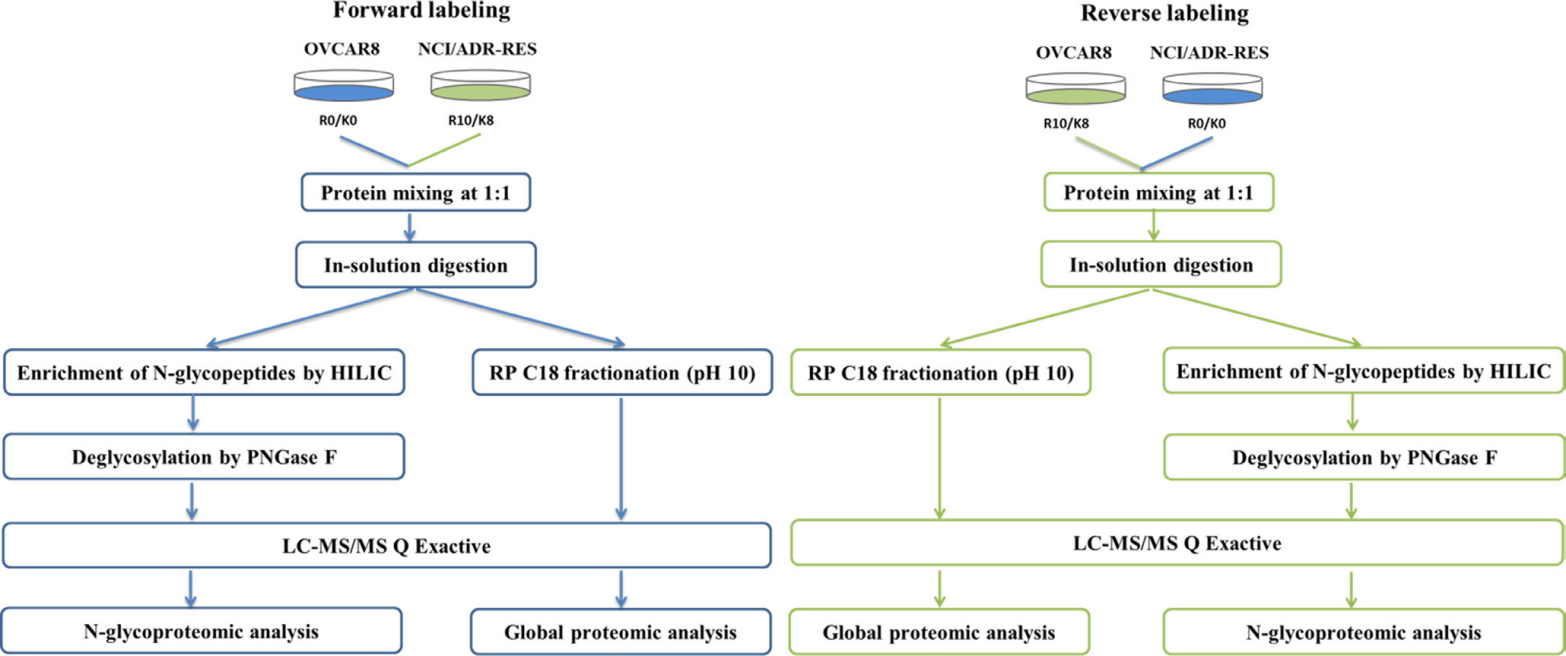
Workflow for the integrated analysis of global protein expression and glycosite-containing peptides In forward labeling experiments, equal amounts of protein obtained from “heavy labeled” NCI/ADR-RES and “light labeled” OVCAR8 cells were combined and followed by in-solution tryptic digestion. Mixed labeled peptide samples were then separated into two aliquots. One aliquot was enriched for glycosite-containing peptides using HILIC and the other aliquot was used for high pH reverse phase C18 fractionation followed by the analysis of global protein expression. Finally, LC-MS/MS analysis of peptides were performed using a LC-MS/MS system. In reverse labeling experiments, “heavy labeled” OVCAR8 and “light labeled” NCI/ADR-RES cells were treated in parallel using procedures same to those described above. Forward labeling and reverse labeling experiments were conducted twice using independent biological replicates.

Figure 1 shows the workflow for the integrated comparative analyses of global proteome and N-glycoproteome of OVCAR8 and its doxorubicin-resistant NCI/ADR-RES cells. To obtain reliable quantitative results, SILAC experiments were conducted in quadruplicate, including two forward and two reverse labeling experiments. For the comparative N-glycoproteomic analysis, a total of 12 samples, three equal amounts of tryptic peptides in each SILAC labeling experiment were enriched for N-glycopeptides by HILIC, removed N-glycans by PNGase F, and analyzed on LC-MS/MS. A total of 1550 N-glycosites were identified, which mapped to 749 protein groups (Supplementary Table 5A). The average Andromeda score of the best identified N-glycosite-containing peptides was 108 and the average localization probability of the glycosylation site to a single amino acid was 98.9%. N-glycosites with a localization probability greater than 0.75 (class Ι sites) and a score difference greater than 5.0 to the next best matching peptide were chosen. Our analyses resulted in 1525 very high confidence sites mapped to 740 protein groups with average localization probability of 99.6%, including 1296, 1332, 1283 and 1197 N-glycosites identified in two forward and two reverse SILAC experiments separately (Supplementary Table 5B). 67.9% of the N-glycosites were identified in all the four replicates (Supplementary Figure 2) and 86.0% (1312 sites) of the N-glycosites were quantified with at least two valid ratios (Supplementary Figure 3, Supplementary Table 5C), indicating a good reproducibility and precision of our quantitative strategy. The spontaneous asparagine (Asn) deamidation has been reported previously [27, 28]. Therefore, we performed an additional control to determine the false positive assignment of N-glycosites. Aliquots of enriched glycopeptides were analyzed directly by LC-MS/MS without PNGase F treatment. Potential false positives were assigned to the samples without PNGase F only if the deamidated Asn was found to be part of the N-linked motif (N-!P-S/T/C). A total of 2 peptides containing deamidated Asn residues and satisfying the N-X-S/T/C sequon were identified (Supplementary Table 6), giving rise to a 0.13% false discovery rate. The false positive rates correlated closely with the enrichment specificities. Due to the high specificities in this study, few non-glycopeptides containing Asn residues were present in the samples, and even fewer contained the N-!P-S/T/C motif. So these N-glycosites which we obtained using our optimized IP-ZIC-HILIC method are highly confident. Finally, 1525 very high confidence N-glycosites were used in further qualitative analysis and 1312 N-glycosites with a minimum of two valid ratios were used in further quantitative analysis.

**Figure 2:**
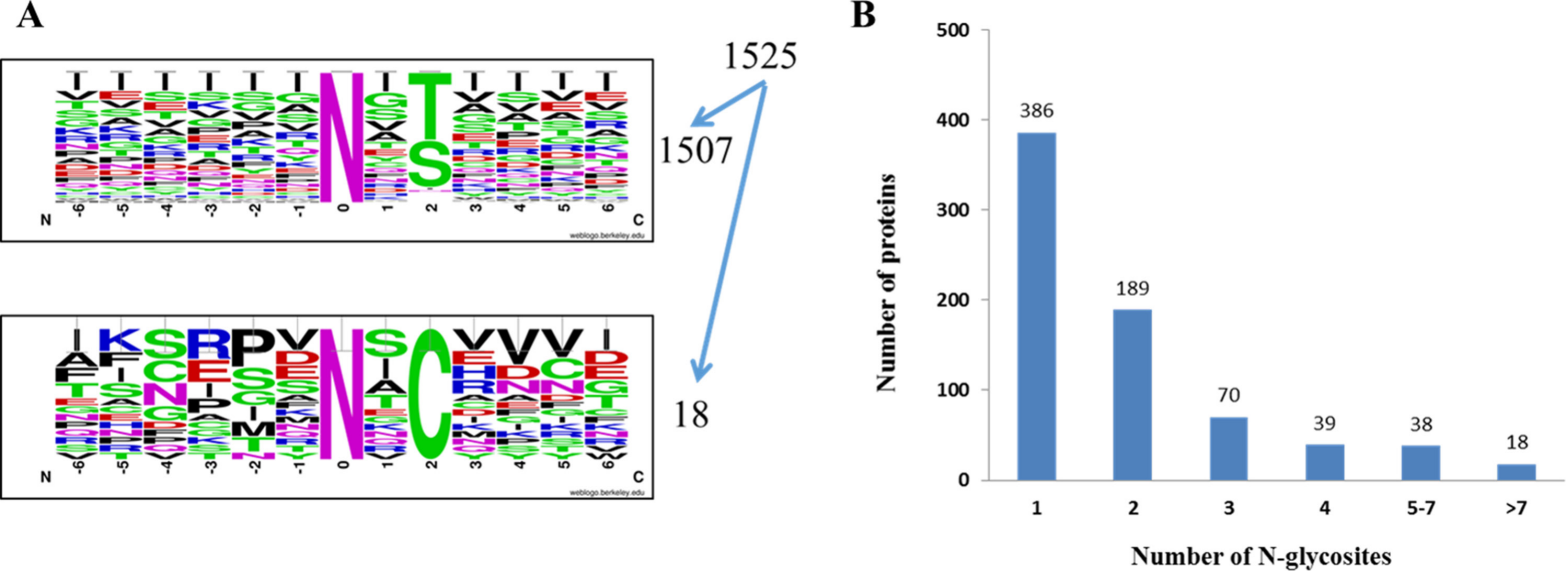
OVCAR8 and NCI/ADR-RES cells N-glycosites identified **(A)** Motif analysis of 1525 class Ι glycosites identified. **(B)** Number of N-glycosylation sites identified per protein.

In the qualitative analysis, we totally identified 1525 unique N-glycosites that matched with the N-glycosylation consensus sequence motif (N-X-S/T/C; X≠P) [29–31]. To be specific, 58.2% (888 sites) of the glycosites match the N-X-T motif, 40.6% (619 sites) of the glycosites match the N-X-S motif and 1.2% (18 sites) of the glycosites match the N-X-C motif (Figure 2A). Notably, Threonine occurs more frequently (1.4-fold) than serine at the second position. The website-based programs Motif-X [32] and WebLogo were used to characterize the motif of identified N-glycosylation sites. These results are in agreement with previous reports [26].

Matching our data set to the UniProtKB database (release in 2015.10) shows that among the 740 N-glycoproteins we identified, 54.2% (401 proteins) are annotated as glycoproteins, and the rest of 339 proteins are indicated as novel. Figure 2B shows the distribution of the number of N-glycosylation sites identified per protein. 386 glycoproteins were identified with a single N-glycosite, and 18 glycoproteins were identified with more than seven N-glycosites. Low density lipoprotein receptor-related protein 1 (LRP1), which plays a vital role in endocytosis and phagocytosis of apoptotic cells, was identified with the maximum of 18 N-glycosites. Lysosome associated membrane glycoprotein 1 (LAMP1) and Lysosome associated membrane glycoprotein 2 (LAMP2), which implicates in tumor cell metastasis, were identified with 12 and 9 N-glycosites separately.

**Figure 3:**
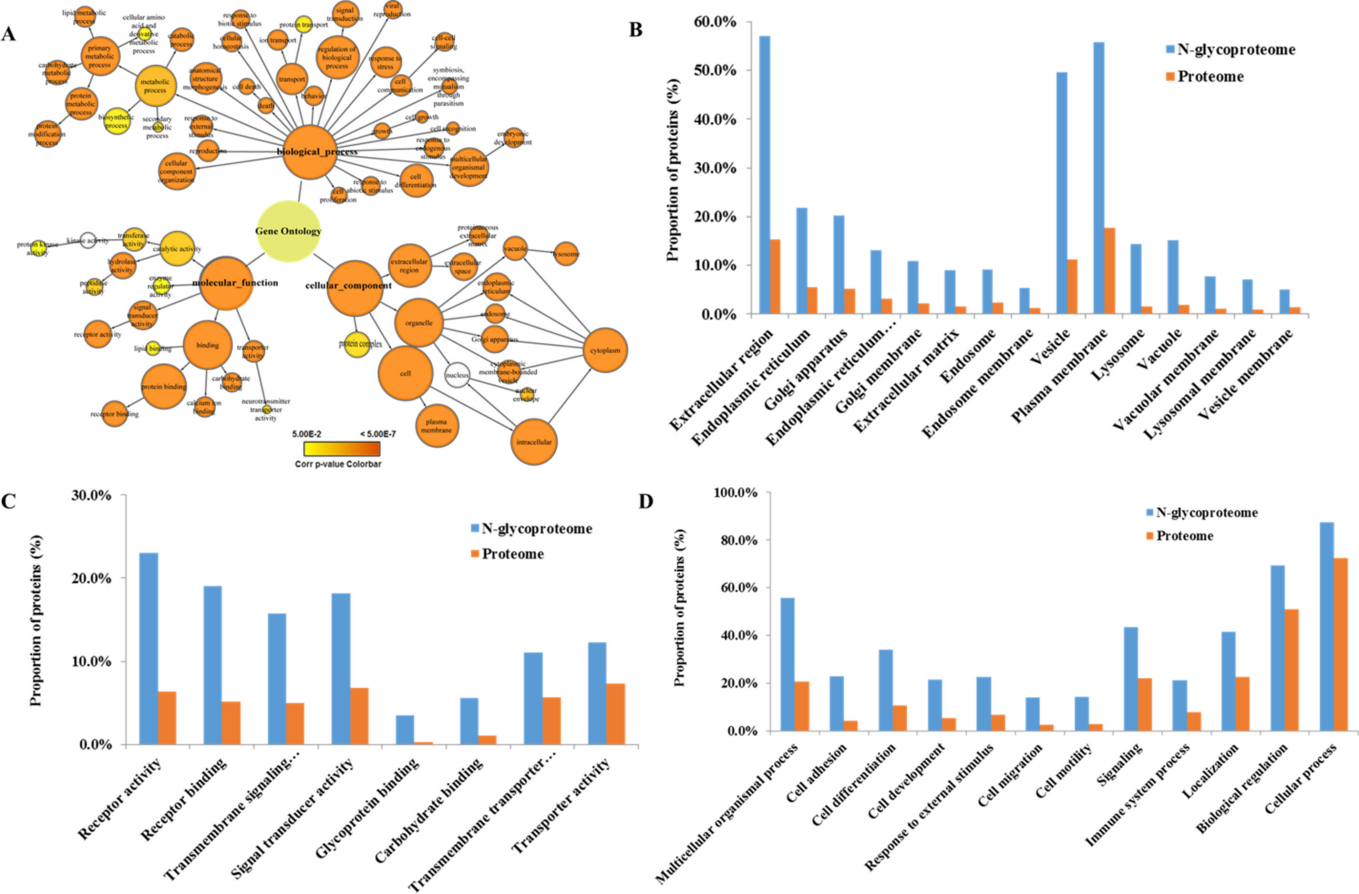
Gene ontology analysis **(A)** GOSlim Generic assignment provided an initial overview of the GO distribution. **(B)** Cellular components are significantly overrepresented in the N-glycoproteome compared to the entire human proteome. **(C)** Molecular functions that are significantly overrepresented in the N-glycoproteome compared to the entire human proteome. **(D)** Biological processes that are significantly overrepresented in the N-glycoproteome compared to the entire human proteome.

To obtain a general overview of the cellular component, molecular function, and biological processes of these identified glycoproteins, we used Cytoscape and BiNGO to determine Gene Ontology (GO) categories overrepresented in the 740 protein groups compared to the entire human proteome. A GOSlim Generic assignment provided an initial overview of the GO distribution (Figure 3A, Supplementary Table 7A). About 56% of the glycoproteome was in the “plasma membrane” GO category (*p* = 0.00E+00, 723 of 740 protein groups with GO annotation). Moreover, proteins associated with the endoplasmic reticulum, Golgi apparatus, lysosome, endosome and vacuole were also highly overrepresented (*p <* 6.20E-20)(Figure 3B, Supplementary Table 7B). Molecular function categories which are enriched in our dataset include receptor activity, transporter activity, receptor binding and carbohydrate binding (*p <* 5.46E 05). These functions are known to be characteristics of glycoproteins and correlate with their extracellular location (Figure 3C, Supplementary Table 7C). The glycoproteome were enriched in many biological processes, that play vital roles in the development of chemoresistance and cancer progression, including cell adhesion, migration, development and differentiation (*p <* 9.88E-38), response to external stimuli (*p* < 1.93E-40) and multicellular organismal processes (*p <* 2.63E-88) (Figure 3D, Supplementary Table 7D).

**Figure 4:**
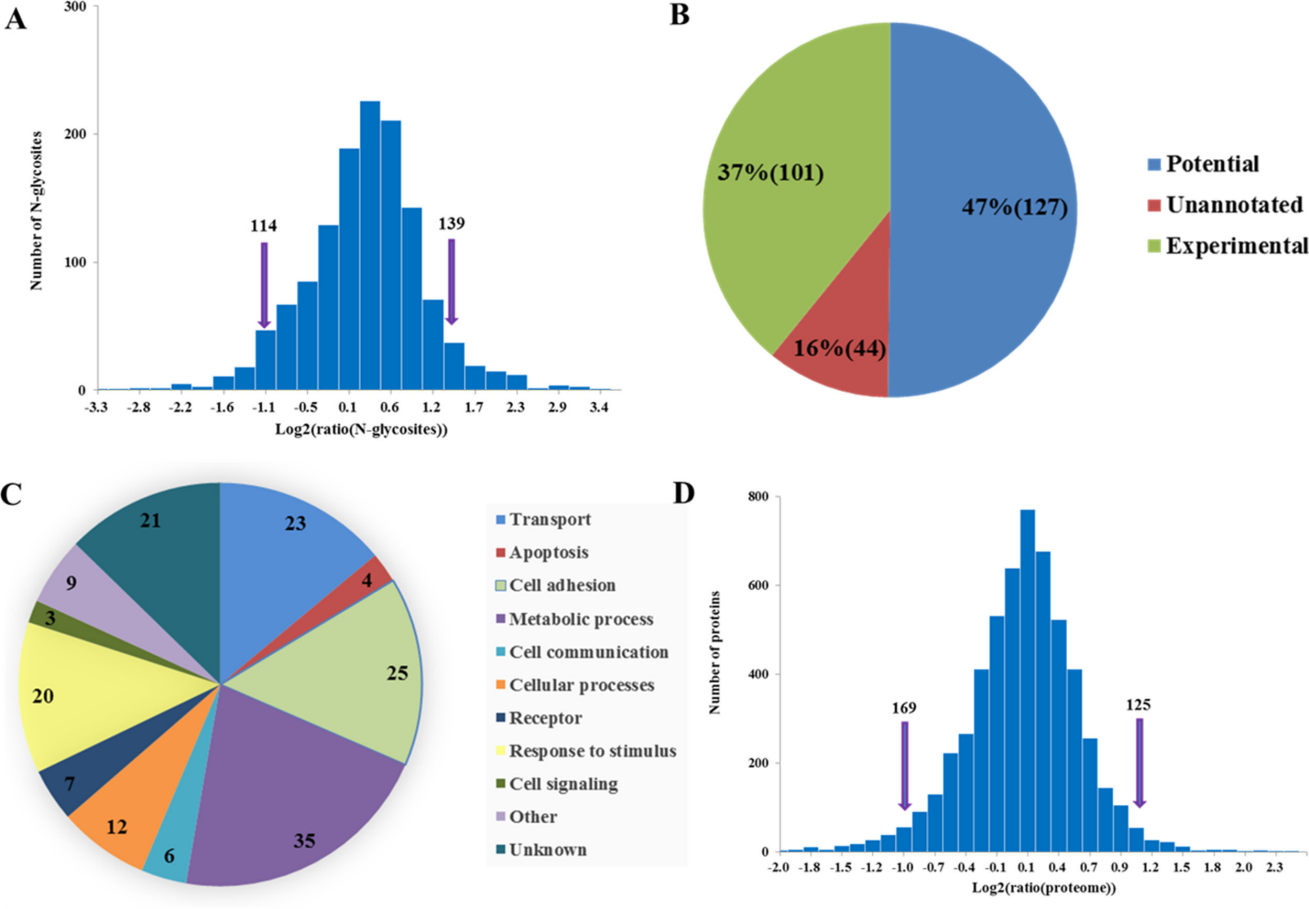
Comparative analyses of N-glycosylation level and proteome between NCI/ADR-RES and OVCAR8 cells **(A)** Log2 ratio distributions for N-glycosites analyzed, note that greater than 1 or less than −1 were considered significant. **(B)** Significantly changed N-glycosites (253) match to Uniprot database annotations. **(C)** Functional annotation of glycoproteins with significantly changed N-glycosite-containing peptides. **(D)** Log2 ratio distributions for proteome analyzed, note that greater than 1 or less than −1 were considered significant.

In the quantitative analysis of N-glycosite-containing peptides, two fold differences were chosen as the threshold for high significance. With these criteria, 114 N-glycosite-containing peptides were observed significantly down-regulated and 139 N-Linked glycosite-containing peptides were observed significantly up-regulated in doxorubicin-resistant NCI/ADR-RES cells compared with OVCAR8 cells (Figure 4A). Among the 253 significantly changed N-glycosite-containing peptides, 226 of them (89%) are annotated to be glycosylated in the UniprotKB database (release in 2015.10), including 127 predicted or based on similarity, and confirmed in our study (Figure 4B, Supplementary Table 8). A total of 165 glycoproteins with 253 significantly changed N-glycosites were analyzed using the PANTHE software [33–35] and UniProtKB database to acquire the information of their functions. Most of these glycoproteins were related to substance metabolism, cell apoptosis, cell adhesion, cell signaling, receptors and transport, indicating that multiple pathways are involved in doxorubicin resistance in ovarian cancer cells (Figure 4C, Supplementary Table 9).

### Global proteomic analysis in OVCAR8 and NCI/ADR-RES cells

At the same time, we also systematically compared the proteome of OVCAR8 and NCI/ADR-RES cells (Figure 1).

SILAC-based quantitative proteomic analyses led to identification of 7101 protein groups with at least one unique peptide identified per protein (Supplementary Table 10A) and quantification of 5509 protein groups with at least two valid ratios were considered for further analysis (Supplementary Table 10B). 76% of the protein groups were identified in all the four replicates (Supplementary Figure 4), and 78% of the protein groups were quantified availably at least twice in the four replicates (Supplementary Figure 5), demonstrating a good reproducibility and accuracy in LC-MS/MS analysis. The mean value of 5509 protein groups quantitative ratios was 1.11 and SD value was 0.20, indicating expression level of most of proteins were unchanged, thus two fold differences were considered to be significant changes of protein expression. Drawing on these criteria, 169 protein groups were down-regulated and 125 protein groups were up-regulated in NCI/ADR-RES cells (Figure 4D). In addition, the accuracy of SILAC-based quantification was validated using western blot. Western blot analysis of OAT, PHB, VDAC1, ATP5B and HINT2 expression in the two cell lines displayed very good consistency with SILAC-based quantification results (Supplementary Figure 6).

Above quantified 5509 proteins include 449 annotated glycoproteins and 70 glycosyltransferases. Among the 449 glycoproteins, 18 glycoproteins including multidrug resistance protein 1 (ABCB1), semaphorin-3D (SEMA3D), transferrin receptor protein 1 (TFRC) and integrin beta-4 (ITGB4) were up-regulated, and 25 glycoproteins were down-regulated at the protein expression level in NCI/ADR-RES cells compared with OVCAR8 cells (Supplementary Table 11).

### The proteome versus the N-glycoproteome

**Figure 5:**
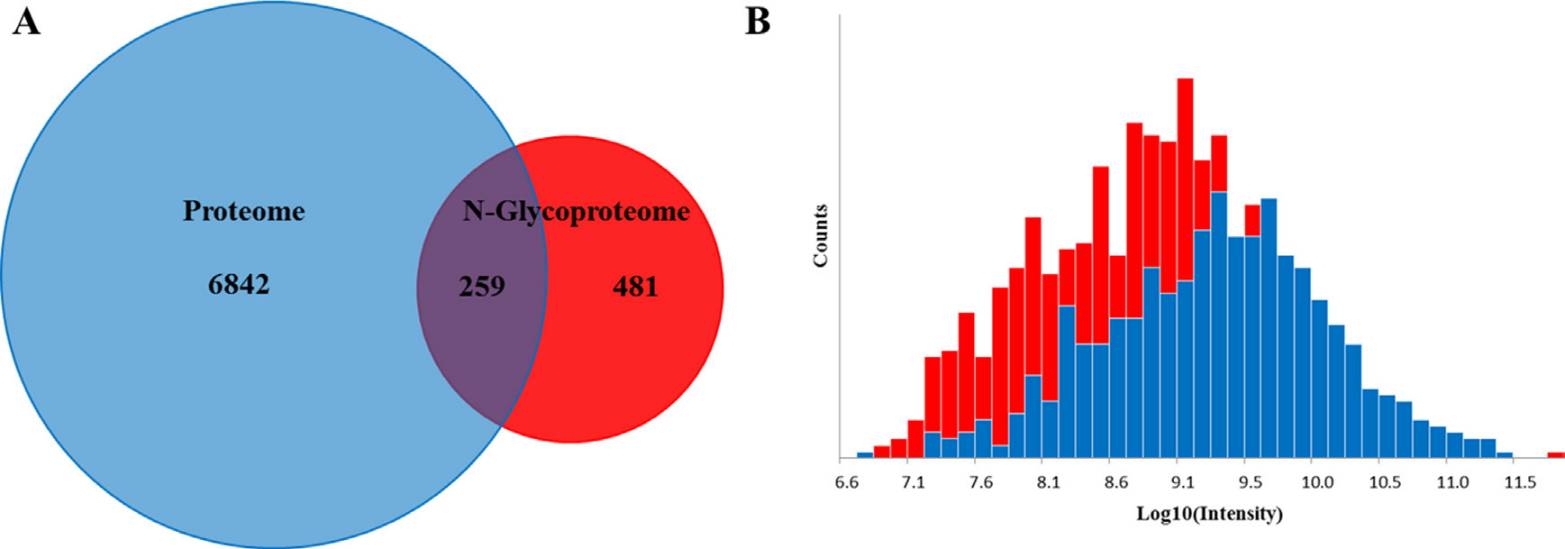
Global proteome versus the N-glycoproteome **(A)** The overlap in the protein groups identified in both proteome and N-glycoproteome. **(B)** Intensity distribution of all glycosites only identified in the N-glyco experiment (red bars) overlaid with intensity distribution of N-glycosites whose corresponding proteins were also identified in the proteome data set (blue bars).

In order to investigate how the abundance of N-glycosite compares to that of proteins, we compared datasets of the quantified N-glycoproteome (N-glycosite-containing peptides and proteins) and the proteome. Figure 5A shows the overlap in the protein groups identified in both proteome and N-glycoproteome. There are 259 proteins identified in both proteome N-glycoproteome. Remarkably, 481 proteins were only identified by their N-glycosylated peptides in the N-glycoproteome. Most soluble and membrane-bound proteins expressed in the endoplasmic reticulum, including secreted proteins, surface receptors and ligands and organelle-resident proteins, were glycosylated to some extent. Nevertheless, their relatively low abundance makes them more difficult to detect in the proteome. This indicated that some low abundant membrane proteins were enriched and identified using N-glycoproteomic approach. This is also supported by the fact that the intensities of de-glycopeptides of proteins only identified in the N-glycoproteome experiment are shifted to low intensity values compared with those where the corresponding proteins were also identified in the global proteome data set (Figure 5B).

Mass spectrometry (MS) based quantitative proteomic techniques have been widely used in simultaneous identification and relative quantification of post-translational modification (PTM) events [36–39]. Though such studies have provided insight into PTM dynamics, we need to interpret the quantitative data carefully because differential abundances of PTM's peptides represent not only changes in PTM's status, but also modified expression of substrate proteins. Recently, researchers tried to decipher the roles of protein abundances playing in the PTM's peptide quantitative data, especially focused on glycosylation [40, 41] and phosphorylation events [42–45]. Gygi et al. reported that correct interpretation of comprehensive phosphorylation dynamics requires normalization by protein expression changes, and he showed an obviously different view, with 25% of seemingly differential phosphopeptides attributed to changes in protein expression [46].

**Figure 6:**
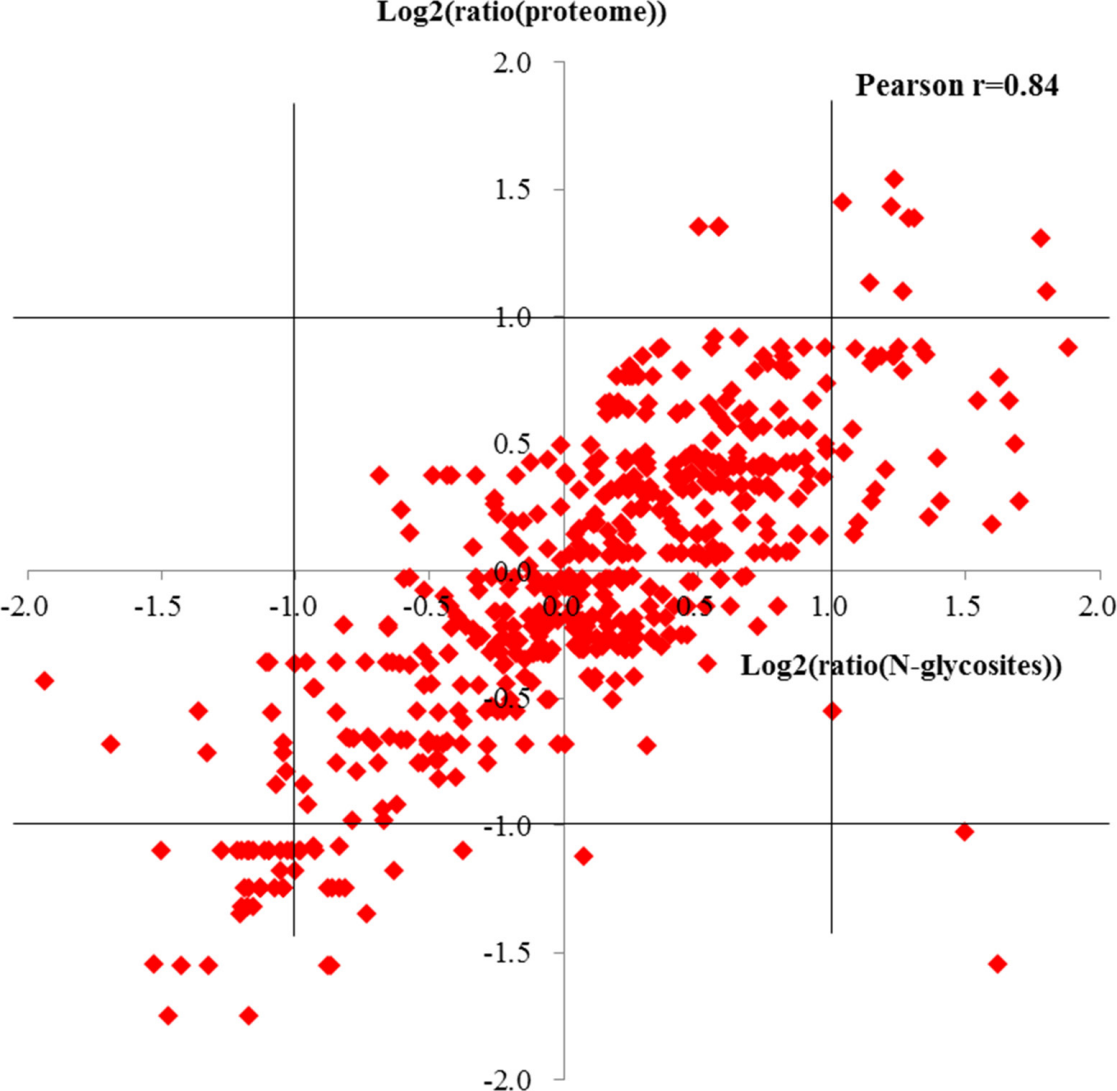
Pearson correlations of glycosites ratios against protein ratios

To evaluate the effect of protein normalization on measured glycosylation changes, we focused on the N-glycoproteins quantified in both proteome and N-glycoproteome, and compared the quantitative N-glycoproteomic ratios of 570 N-glycosites which mapped to 208 proteins, and the ratios in protein expression level after filtering with at least two valid ratio values (Supplementary Table 12). The ratios of the N-glycosites against protein ratios correlate well (Pearson *r* = 0.84), indicating that changes in N-glycosylation of a protein are usually a reflection of the change of protein abundance. However, protein ratios were located within a range of ±1 (log_2_ scale) and glycosylation site ratios were outside the –1 and +1 interval at the same time suggesting significant changes in the glycosylation occupancy of these proteins (Figure 6).

**Figure 7:**
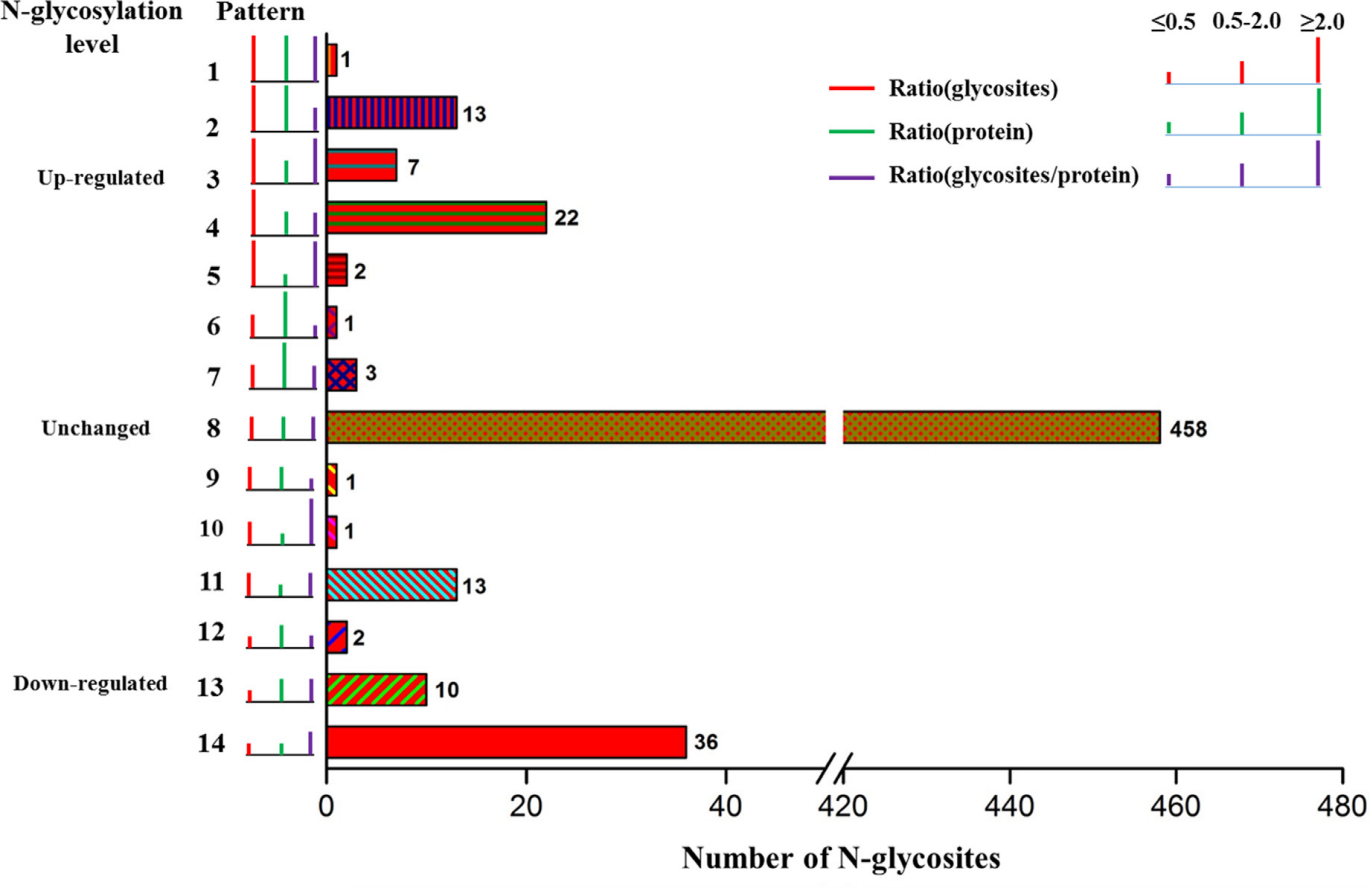
The patterns of N-glycosite-containing peptides changes before and after normalization by protein abundance

To be specific, in order to obtain the actual glycosylation change of every protein, 570 N-glycosites were classified into 14 patterns according to N-glycosites ratio, protein ratio and calibrated N-glycosites ratio (so called N-glycosites occupancy) (Figure 7). Briefly, the 570 N-glycosites were firstly grouped into three major categories based on N-glycosites ratio: up-regulated (≥ 2.0), down-regulated (≤ 0.5) and unchanged (0.5–2.0). Within each category, the N-glycosites were further sorted into different subcategories according to their protein expression ratio and calibrated N-glycosites ratio. Pattern 1 (P1), pattern 3 (P3) and pattern 5 (P5) displayed that N-glycosite-containing peptides were significantly up-regulated before and after calibration, indicating that these N-glycosite-containing peptides changes were indeed resulted from increased N-glycosylation occupancy. In P3, for example, actually up-regulated N-glycosylation level of ATP6AP1 at amino acid residues Asn261 provided additional information on interpreting the role of ATP6AP1 in drug resistance of cancers. [47, 48] Pattern 2 (P2), pattern 4 (P4), pattern 13 (P13) and pattern 14 (P14) showed that the significantly up- or down-regulated N-glycosite-containing peptides were found unchanged N-glycosite occupancy after calibration using protein expression ratios. In P2, both of N-glycosylation level of ABCB1 at Asn99 and its protein expression were extremely up-regulated, and the actual N-glycosite occupancy at Asn99 was unchanged after calibration, so the effect of ABCB1 glycosylation on its drug efflux activity needs to be validated by more studies [12, 49–52]. Pattern 6 (P6), pattern 9 (P9) and pattern 10 (P10) revealed that these significantly changed N-glycosite-containing peptides with unchanged N-glycosite occupancy. Pattern 7 (P7), pattern 8 (P8) and pattern 11 (P11) showed that 458 N-glycosite-containing peptides had unchanged N-glycosylation level and N-glycosite occupancy. The representative N-glycosite-containing peptides and their classification were listed in Table 1.

**Table 1:** Representative lists of N-glycosite-containing peptides whose changes before and after normalization by protein abundance

Pattern	Protein	Gene names	Deamidation (N) Probabilities	Position	Glycopeptide ratio	Protein ratio	Calibrated ratio
1	P03956	MMP1	AFQIWSN(1)VTPITFTK	143	6.07 ± 2.32	2.14 ± 0.92	2.84
2	O75326-2	SEMA7A	HPSCWNIVN(1)GTVVPIGEMR	143	2.41 ± 0.88	2.15 ± 0.67	1.12
			NPEAPIN(1)VSR	244	3.48 ± 2.10	2.15 ± 0.67	1.62
	B5MCA4	EPCAM	QCN(1)GTSMCWCVNTAGVR	139	2.21 ± 0.26	2.19 ± 0.09	1.01
	Q9H9S5	FKRP	DIFN(1)ISAPIAR	209	3.43 ± 0.76	2.48 ± 1.68	1.38
	A0A087X0S5	COL6A1	N(1)VTAQICIDK	802	2.44 ± 0.06	2.62 ± 0.94	0.93
			RN(1)FTAADWGQSR	212	2.47 ± 0.90	2.62 ± 0.94	0.95
	Q9H6B4	CLMP	HVYN(1)N(1)ITEEQK	74	2.34 ± 0.29	2.70 ± 0.57	0.86
	F6SFZ6	P2RX4	CVAFN(1)GSVK	153	2.06 ± 0.44	2.73 ± 0.19	0.75
	Q06481-5	APLP2	RN(1)QSISIIYK	312	2.35 ± 0.17	2.91 ± 0.39	0.81
	P17813-2	ENG	QN(1)GTWPR	88	7.47 ± 1.13	5.92 ± 2.58	1.26
	H0Y5C0	ADGRL2	GPDISN(1)CTSHWVNQIAQK	388	7.13 ± 1.55	6.62 ± 2.45	1.08
	O95025	SEMA3D	VIQPYN(1)K	139	94.03 ± 66.77	48.82 ± 60.86	1.93
	P08183	ABCB1	SDIN(1)DTGFFMNIEEDMTR	99	92.41 ± 59.54	56.46 ± 29.53	1.64
3	P32004-3	L1CAM	FFPYAN(1)GTIGIR	474	2.00 ± 0.71	0.68 ± 0.09	2.93
	Q15904	ATP6AP1	QPVSPVIHPPVSYN(1)DTAPR	261	3.03 ± 2.84	1.14 ± 0.06	2.67
	O43852-5	CALU	N(1)ATYGYVIDDPDPDDGFNYK	131	2.57 ± 0.87	1.16 ± 0.14	2.22
	G3XAI2	LAMB1	MEMPSTPQQIQN(1)ITEDIR	1566	2.64 ± 1.70	1.21 ± 0.17	2.19
			GTN(1)YTVR	701	3.24 ± 0.13	1.21 ± 0.17	2.68
	Q76M96	CCDC80	IIGVGEEVGGVIEIFPIN(1)GSSVVEREDVPAHIVK	835	3.21 ± 1.29	1.42 ± 0.25	2.26
	P15144	ANPEP	GPSTPIPEDPNWN(1)VTEFHTTPK	265	3.68 ± 2.96	1.84 ± 0.18	2.00
4	C9J5X1	IGF1R	N(1)GSQSMYCIPCEGPCPK	314	2.11 ± 1.03	1.11 ± 0.39	1.91
	Q6UY14	ADAMTSL4	IGTEFN(1)VTSPSN(1)CSHIPRPPAIQPCQGQACQDR	946	2.14 ± 1.66	1.14 ± 0.36	1.88
			IGTEFN(1)VTSPSN(1)CSHIPRPPAIQPCQGQACQDR	952	2.14 ± 1.66	1.14 ± 0.36	1.88
	G3XAI2	LAMB1	ISDTTSQSN(1)STAK	1303	2.21 ± 0.32	1.21 ± 0.17	1.83
	C9JP16	CRTAP	N(1)CSAAPQPEPAAGIASYPEIR	87	2.24 ± 1.65	1.25 ± 0.12	1.80
	Q15293	RCN1	VVRPDSEIGERPPEDN(1)QSFQYDHEAFIGK	53	2.30 ± 1.64	1.32 ± 0.06	1.74
	Q96L58	B3GALT6	DAYEN(1)ITAK	131	2.63 ± 0.28	1.36 ± 0.91	1.93
	Q02809	PLOD1	REQIN(1)ITIDHR	197	2.06 ± 0.56	1.38 ± 0.22	1.49
	A0A0A0MSA9	PVR	VEDEGN(1)YTCIFVTFPQGSR	120	2.11 ± 0.39	1.47 ± 0.20	1.43
	P25391	LAMA1	HQVSIN(0.979)N(0.021)TAVMQR	555	2.91 ± 0.81	1.60 ± 0.52	1.83
			DVAGISQEIIN(1)TSASISR	2038	3.17 ± 1.02	1.60 ± 0.52	1.99
	Q9H488-2	POFUT1	EGNPFGPFWDQFHVSFN(1)K	160	3.08 ± 1.13	1.69 ± 0.30	1.82
	Q92859-3	NEO1	TISDVPSAAPQN(1)ISIEVR	639	2.40 ± 0.94	1.73 ± 0.42	1.39
	O15440-5	ABCC5	QGSGN(1)TTVTRGN(1)ETSVSDSMK	897	2.21 ± 0.41	1.76 ± 0.09	1.26
	P78324-4	SIRPA	IQITWIEN(1)GN(1)VSR	291	2.23 ± 0.31	1.80 ± 0.65	1.24
			IGN(1)ITPADAGTYYCVK	110	2.27 ± 0.29	1.80 ± 0.65	1.26
			GTAN(1)ISETIR	244	2.34 ± 0.40	1.80 ± 0.65	1.30
			AENQVN(1)VTCQVR	269	2.35 ± 0.49	1.80 ± 0.65	1.30
	Q17RY6	LY6K	YCN(1)IEGPPIN(1)SSVFK	129	2.55 ± 0.85	1.80 ± 0.14	1.42
	A0A087WZ85	ROBO1	NYIGEAVSHN(1)ASIEVAIIR	41	2.13 ± 0.43	1.83 ± 0.33	1.16
	P43121	MCAM	ENMVIN(1)ISCEASGHPR	449	2.37 ± 0.15	1.84 ± 0.33	1.29
			CVASVPSIPGIN(1)R	418	2.52 ± 0.79	1.84 ± 0.33	1.37
5	P56159-2	GFRA1	ETN(1)FSIASGIEAK	59	3.07 ± 0.55	0.34 ± 0.11	8.95
	C9JE12	TMUB1	FIN(1)DSEQVAR	111	2.82 ± 1.99	0.49 ± 0.25	5.73
6	H0Y5C0	ADGRL2	SIGQFISTEN(1)ATIK	599	1.83 ± 0.19	6.62 ± 2.45	0.28
9	P40189-3	IL6ST	ETHIETN(1)FTIK	157	0.62 ± 0.24	1.30 ± 0.43	0.48
10	Q96L35	EPHB4	IN(1)GSSIHIEWSAPIESGGR	335	1.05 ± 0.92	0.46 ± 0.06	2.28
12	P26006	ITGA3	DDCERMN(1)ITVK	107	0.31 ± 0.01	0.63 ± 0.06	0.50
	Q16610	ECM1	QGN(0.04)N(0.96)HTCTWK	354	0.26 ± 0.09	0.74 ± 0.17	0.35
13	Q92508	PIEZO1	HMIAIAPN(1)STAR	2331	0.48 ± 0.13	0.56 ± 0.10	0.85
	O60568	PLOD3	EQYIHEN(1)YSR	548	0.49 ± 0.08	0.58 ± 0.06	0.84
	H0YNA7	SPPL2A	DMN(1)QTIGDN(1)ITVK	107	0.40 ± 0.10	0.61 ± 0.11	0.65
			DMN(1)QTIGDN(1)ITVK	113	0.48 ± 0.06	0.61 ± 0.11	0.80
	P08473	MME	EIAN(1)ATAKPEDR	285	0.49 ± 0.06	0.63 ± 0.01	0.77
	A0A0A0MT32	LIPA	YDIPASINFIIN(1)K	45	0.47 ± 0.07	0.68 ± 0.03	0.69
	P32004-3	L1CAM	GYN(1)VTYWR	844	0.39 ± 0.03	0.68 ± 0.09	0.57
	K7ELL7	PRKCSH	YEQGTGCWQGPN(1)R	483	0.50 ± 0.05	0.78 ± 0.04	0.64
	P56199	ITGA1	YN(1)HTGQVIIYR	460	0.46 ± 0.16	0.78 ± 0.21	0.59
			SEN(1)ASIVISSSNQK	1113	0.47 ± 0.08	0.78 ± 0.21	0.60
14	P43007	SLC1A4	VVTQN(1)SSSGN(1)VTHEK	206	0.23 ± 0.06	0.24 ± 0.05	0.95
			VVTQN(1)SSSGN(1)VTHEK	201	0.30 ± 0.05	0.24 ± 0.05	1.22
	P32970	CD70	GDTICTN(1)ITGTIIPSR	170	0.36 ± 0.07	0.30 ± 0.08	1.21
			FAQAQQQIPIESIGWDVAEIQIN(1)HTGPQQDPR	63	0.44 ± 0.08	0.30 ± 0.08	1.49
	Q99523	SORT1	IAN(1)N(1)THQHVFDDIR	98	0.37 ± 0.06	0.34 ± 0.06	1.09
			DITDIIN(1)N(1)TFIR	162	0.40 ± 0.08	0.34 ± 0.06	1.17
	P56159-2	GFRA1	SN(0.999)VSGN(0.002)THICISN(0.736)GN(0.262)YEK	401	0.35 ± 0.06	0.34 ± 0.11	1.01
	Q6UW63	KDELC1	N(1)STAVWR	302	0.43 ± 0.05	0.39 ± 0.06	1.10
	Q68CQ7	GLT8D1	RQN(1)ITNQIEK	257	0.44 ± 0.04	0.40 ± 0.03	1.10
			SNVIFYIVTIN(0.998)N(0.002)TADHIR	103	0.45 ± 0.07	0.40 ± 0.03	1.12
	Q495W5-2	FUT11	IFN(1)ITSTFSR	166	0.44 ± 0.08	0.40 ± 0.04	1.08
	P11279	LAMP1	IININ(0.007)PN(0.993)K	261	0.44 ± 0.04	0.42 ± 0.03	1.04
			GHTITIN(1)FTR	103	0.44 ± 0.12	0.42 ± 0.03	1.05
			SPSVDKYN(1)VSGTN(1)GTCIIASMGIQIN(1)ITYER	223	0.46 ± 0.10	0.42 ± 0.03	1.08
			SPSVDKYN(1)VSGTN(1)GTCIIASMGIQIN(1)ITYER	228	0.46 ± 0.10	0.42 ± 0.03	1.08
			SPSVDKYN(1)VSGTN(1)GTCIIASMGIQIN(1)ITYER	241	0.46 ± 0.10	0.42 ± 0.03	1.08
			N(1)MTFDIPSDATVVINR	62	0.47 ± 0.05	0.42 ± 0.03	1.12
			YSVQIMSFVYN(1)ISDTHIFPN(1)ASSK	121	0.48 ± 0.18	0.42 ± 0.03	1.15
			YSVQIMSFVYN(1)ISDTHIFPN(1)ASSK	130	0.48 ± 0.18	0.42 ± 0.03	1.15
	Q10589-2	BST2	GFQDVEAQAATCN(1)HTVMAIMASIDAEK	80	0.48 ± 0.07	0.44 ± 0.07	1.09
			N(1)VTHIIQQEITEAQK	53	0.50 ± 0.09	0.44 ± 0.07	1.13
	Q07954	LRP1	DN(1)TTCYEFKK	1575	0.35 ± 0.09	0.47 ± 0.07	0.76
			TCVSN(1)CTASQFVCK	3333	0.41 ± 0.16	0.47 ± 0.07	0.88
			GVTHIN(1)ISGIK	3953	0.43 ± 0.11	0.47 ± 0.07	0.92
			MHIN(1)GSNVQVIHR	3089	0.43 ± 0.10	0.47 ± 0.07	0.93
			CN(1)ASSQFICSSGR	2905	0.44 ± 0.04	0.47 ± 0.07	0.94
			IETIIIN(1)GTDRK	729	0.44 ± 0.03	0.47 ± 0.07	0.95
			WTGHN(1)VTVVQR	1511	0.44 ± 0.13	0.47 ± 0.07	0.95
			IN(1)GTDPIVAADSK	4075	0.45 ± 0.05	0.47 ± 0.07	0.96
			INIDGSN(1)YTIIK	3048	0.46 ± 0.05	0.47 ± 0.07	0.99
			IYWTDGDN(1)ISMAN(0.002)MDGSN(0.998)R	1733	0.47 ± 0.09	0.47 ± 0.07	1.00
			IYWTDGDN(1)ISMAN(0.002)MDGSN(0.998)R	1723	0.47 ± 0.09	0.47 ± 0.07	1.00
			FGTCSQICN(0.996)N(0.004)TK	3839	0.48 ± 0.11	0.47 ± 0.07	1.03
			ITSCATN(1)ASICGDEAR	3788	0.49 ± 0.10	0.47 ± 0.07	1.05
			FN(1)STEYQVVTR	446	0.50 ± 0.09	0.47 ± 0.07	1.08
	P18465	HLA-B	YYN(1)QSEAGSHIIQVMYGCDVGPDGR	110	0.49 ± 0.05	0.47 ± 0.03	1.06

## DISCUSSION

OVCAR8 and its doxorubicin-resistant NCI/ADR-RES cells have been used models to study the mechanisms of multidrug resistance. Our previous study has compared the differentially expressed mitochondria proteome between OVCAR8 and NCI/ADR-RES cells using SILAC-based quantitative proteomic strategy, and several annotated glycoproteins, such as multidrug resistant protein, fukutin-related protein, adhesion G protein-coupled receptor L2 and CXADR-like membrane protein, have been found up-regulated in doxorubicin-resistant NCI/ADR-RES cells [53]. In this study, we have provided the most comprehensive comparative proteomic and N-glycoproteomic analyses of OVCAR8 and its doxorubicin-resistant NCI/ADR-RES cells. A total of 7101 protein groups were identified and 5509 protein groups were quantified in the proteomic analysis and 1525 unique N-glycosite-containing peptides from 740 N-glycoproteins were identified and quantified in the N-glycoproteomic analysis. Some low abundant glycosylated membrane proteins were identified and quantified in the N-glycoproteomic analysis, but they were not identified in the global proteomic analysis. The reason is that the glycopeptides from the low abundant membrane proteins have been enriched in the N-glycoproteome analysis. So in total, about 44% (570 of 1312) of unique N-glycosite-containing peptides quantified in N-glycoproteome analysis obtained their corresponding protein ratios from the proteomic analysis. Even so, we are still able to focus on the 570 N-glycosylated sites from 208 proteins, which were quantified in both N-glycoproteomic and proteomic analyses, and compare their N-glycosylation ratios, protein ratios and the calibrated N-glycosylation ratios, also called N-glycosite occupancy (N-glycosylation ratio/protein ratio). The 570 N-glycosites were classified into 14 patterns according to N-glycosites ratio, protein ratio and calibrated N-glycosites ratio.

As expected, N-glycosylation as a largely cotranslational and stable modification, most of the changes in glycoproteins observed at specific N-glycosites were a reflection of the change of protein abundance. However, in some cases, the changes in the N-glycosylation level and the protein level could not maintain consistency. For example, ABCC5, one member of the ABC superfamily, is a multidrug-resistance-associated protein 5, which is related to the drug efflux of multidrug resistance of cancer cells [54–56]. In this study, ABCC5 protein was up-regulated in the N-glycosylation level at Asn897 and unchanged in its protein expression level in the NCI/ADR-RES cells compared with the OVCAR8 cells, where no significant changes were measured in the global expression of the same protein. This study indicated that an overall increase of N-glycosylation occupancy of ABCC5 in drug-resistant NCI/ADR-RES cells. The up-regulated N-glycosylation level of ABCC5 could play a significant role in multidrug resistance of ovarian cancer, but this hypothesis needs to be validated by further studies. Furthermore, P-gp1, the protein product of the ABCB1 (multidrug resistance protein 1), is also responsible for pumping doxorubicin out of cells to reduce the accumulation of doxorubicin in cells [57] and protecting resistant tumor cells from caspase-dependent apoptosis in the mitochondria [58], thus accounting for drug resistance. Here, P-gp1 was found simultaneously up-regulated in the N-glycosylation at Asn99 at a SILAC ratio of 92.41 ± 59.54 and in the expression level at a SILAC ratio of 56.46 ± 29.53 in NCI/ADR-RES cells compared to OVCAR8 cells. This indicated that both of the up-regulated N-glycosylation level and expression level of P-gp1 are related to multidrug resistance of ovarian cancer.

## CONCLUSIONS

In this study, the established HILIC based enrichment method for N-linked glycopeptides are of a good stability, reproducibility and high enrichment specificity. Integrated proteomic and N-glycoproteomic analyses of doxorubicin sensitive and resistant ovarian cells provide the glycoprotein alteration in protein abundance, glycosylation level and N-glycosylation occupancy, which can help to unravel the relationship of N-glycosylation and MDR, understand the mechanism of MDR, and discover the new diagnostic and therapeutic targets.

## MATERIALS AND METHODS

### Cell culture and SILAC labeling

The human ovarian tumor cell line OVCAR8 and its doxorubicin resistant subline NCI/ADR-RES were gifts from Dr. Xiaoyuan (Shawn) Chen of the National Institutes of Health (NCI/ADR-RES is verified as a multidrug resistance cell line by Dr. Chen group [53]). OVCAR8 cells were maintained in RPMI 1640 medium supplemented with 10% heat-inactivated FBS, 2 mM L-glutamine, 100 U/mL penicillin and 100 μg/mL streptomycin in a humidified atmosphere containing 5% CO_2_ at 37°C. Culture condition for NCI/ADR-RES cells were identical with OVCAR8 cells except that the concentration of L-glutamine was 5 mM.

For forward SILAC labeling, OVCAR8 cells were labeled with light amino acid by growing them in RPMI 1640 medium containing L-[^12^C_6_, ^14^N_4_]-arginine (Arg0) and L-[^12^C_6_, ^14^N_2_]-lysine (Lys0), and NCI/ADR-RES cells were labeled with heavy amino acids by growing them in RPMI 1640 medium containing L-[^13^C_6_, ^15^N_4_]-arginine (Arg10) and L-[^13^C_6_, ^15^N_2_]-lysine (Lys8) instead of the natural amino acids. For reverse labeling, the OVCAR8 cells were grown in heavy SILAC medium, and NCI/ADR-RES cells were grown in light SILAC medium. Labeled amino acids were purchased from Cambridge Isotope Laboratories (Andover, MA). For SILAC cell culture, cells were grown for at least six passages in the SILAC medium supplemented with 10% heat-inactivated dialyzed fetal bovine serum (Invitrogen, Carlsbad, CA) to achieve complete isotope incorporation (> 96%).

NCI/ADR-RES cells, which were originally thought to be derived from a MCF-7 breast cancer cell line, were later validated to be derived from OVCAR8 ovarian adenocarcinoma cells [59]. Measurement of doxorubicin resistance by MTT analysis in OVCAR8 and NCI/ADR-RES cells was described previously [53].

### Protein extraction and digestion

Cells were washed three times with cold PBS and then harvested using a cell scraper in lysis buffer (8M urea, 0.1 M Tris-HCl, pH 8.5) containing Protease Inhibitor Cocktail Tablets (Roche, Basel, Switzerland). The lysates were sonicated and the supernatant was carefully collected after centrifugation at 14,000 g at 4°C for 10 min. The concentration of proteins was determined by Bradford assay. Extracted protein samples from heavy labeled NCI/ADR-RES cells and light labeled OVCAR8 cells in forward SILAC labeling, light labeled NCI/ADR-RES cells and heavy labeled OVCAR8 cells in reverse SILAC labeling were combined at a 1:1 ratio separately. In-solution digestion was performed with the following protocol. Protein mixtures were reduced with 10 mM 1,4-dithiothreitol (DTT) at 37°C for one hour, subsequently alkylated in 40 mM iodoacetamide (IAM) for 30 min at room temperature in the dark and then Lys-C was added and digested at 37°C for 6 hours. After diluting the concentration of urea to 1M with 25 mM NH_4_HCO_3_, sequence grade trypsin (Promega, Madison, WI) was added at an enzyme/protein ratio of 1:50 and digested at 37°C overnight. Digestion was stopped by adding formic acid to a final concentration of 1‰. The digested peptide mixtures were collected by centrifugation at 14, 000 g for 10 min. All digested products were desalted on Waters Oasis^®^ HLB solid phase extraction columns.

### The establishment, optimization and evaluation of hydrophilic interaction liquid chromatography (HILIC) based enrichment method for the N-linked glycopeptides

Enrichment was performed on a RIGOL L-3000 Series HPLC system (including L-3120 solvent organizer, L-3220 binary pump, L-3320 autosampler with a 100 μL sample loop, L-3400 column oven, and L-3500 UV-VIS detector). The HILIC-based enrichment method for the N-linked glycopeptides was established, optimized and evaluated using mouse brain tissue sample, OVCAR8 and NCI/ADR-RES cells samples separately. About 200 μg FASP [60] based tryptic digested peptide mixture was dissolved in 100 μL 60% ACN and injected into Ultimate^®^ HILIC Amphion column (4.6×100 mm, 5 μm, 120 Å, Welch Materials (Shanghai), Inc.). The column was eluted using mobile phase A (0.1% TFA in water) and mobile phase B (0.1% TFA in acetonitrile) with a gradient (80% B, 0–20 min; 80–2% B, 20–21 min; 2% B, 21–30 min; 2–80% B, 30–30.1 min; 80% B, 30.1–38 min) at a flow rate of 0.7 mL/min. The glycopeptides containing fractions were detected at 214 nm and collected from 20 to 30 min. The glycopeptides containing fractions were dried under vacuum, then resuspended in 50 μL 50 mM NH_4_HCO_3_ and deglycosylated with PNGase F at 3°C for 2.5 h and dried again for LC-MS/MS analysis. The specificity of the enrichment for N-glycopeptides was calculated using the number of identified N-glycopeptides divided by the number of total peptides identified.

The SILAC labeled and combined peptide mixture from NCI/ADR-RES and OVCAR8 cells was performed the enrichment for N-glycopeptides using the above optimized method and deglycosylated with PNGase F at 37°C for direct LC-MS/MS analysis without fractionation. To evaluate the chemical deamidation in sample processing, we incubated the same SILAC labeled peptide mixture enriched as above described at 37°C without PNGase F in NH_4_HCO_3_ buffer. The samples were then analyzed by LC-MS/MS in parallel.

### Fractionation of peptides using high-pH HPLC for quantitative proteomic analysis

Fractionation of peptide mixture was performed using a RIGOL L-3000 Series HPLC system. About 50 μg peptide mixture was dissolved in 100 μL water (0.1% ammonium hydroxide), then injected into a Xbridge^®^ Peptide BEH C18 column (2.1×150 mm, 3.5 μm, 130 Å, Waters Corporation) and then eluted using mobile phase A (0.1% ammonium hydroxide in water, pH 10) and mobile phase B (0.1% ammonium hydroxide in acetonitrile, pH 10) with a gradient (5–8% B, 0–5 min; 8–18% B, 5–40 min; 18–32% B, 40–62 min; 32–95% B, 62–64 min; 95–95% B, 64–68 min; 95–5% B, 68–69 min; 5–5% B, 69–76 min) at a flow rate of 0.2 mL/min. Peptides were detected at 214 nm, 48 fractions were collected along with the LC-separation in a time-based mode from 3 to 75 min and then concatenated into 12 fractions by combining fractions 1, 13, 25, 37; 2, 14, 26, 38, etc. The combined fractions were then dried under vacuum and stored at –80°C until LC-MS/MS analysis.

### LC-MS/MS analysis

LC-MS/MS analysis was performed on EASY-nLC 1000 HPLC coupled with Q Exactive mass spectrometer (Thermo Scientific). Lyophilized peptides samples were resuspended in 0.1% FA and loaded onto a reverse-phase C18 in-house packed trap column (100 μm ID × 2 cm, 5 μm, Reprosil-Pur C18 AQ, Dr. Maisch GmbH, Germany) and then separated on a reversed-phase C18 in house packed analytical column (75 μm ID×20 cm, 3 μm, Reprosil-Pur C18 AQ, Dr. Maisch GmbH, Germany) using mobile phase A (0.1% FA in water) and mobile phase B (0.1% FA in acetonitrile) with a gradient (5–8% B, 8 min; 8–22% B, 50 min; 22–32% B, 12 min; 32–95% B, 1 min; 95% B, 7 min) at a flow rate of 280 nL/min. The mass spectrometer was operated in positive ion mode with data-dependent acquisition mode. Full scan MS spectra (from m/z 300 to 1600) were acquired in the Orbitrap at a high resolution of 70,000 (m/z 200) with an automatic gain control (AGC) of 3×10^6^ and a maximum injection time of 60 ms. The top 20 precursor ions were selected from each MS full scan with isolation width of 2 m/z in the HCD collision cell with normalized collision energy of 27%. Subsequently, MS/MS spectra were acquired in the Orbitrap with a resolution of 17,500 at m/z 200. The target value was 5 × 10^4^ with a maximum injection time of 80 ms. Ions selected for MS/MS were dynamically excluded for a duration of 40s. For nano electrospray ion source setting: the spray voltage was 2.0 kV, no sheath gas flow, the heated capillary temperature was 320°C. All raw data were viewed in Xcalibur v2.2.

### Data analysis

The N-glycoproteome and the proteome raw data were analyzed using MaxQuant software (version 1.5.2.8) against UniportKB human database (downloaded on October 15, 2015) with 92,013 entries. For N-glycoproteome analysis, the parameters were set as follows: Cysteine carbamidomethylation was set as a fixed modification and N-terminal acetylation, methionine oxidation and asparagine deamidation were set as variable modifications. Leucines were replaced by isoleucines. Enzyme specificity was set to trypsin allowing N-terminal cleavage to proline. False discovery rate, determined by using a reversed database, was set to 0.01 for peptide, modification site and protein identifications. The minimum required peptide length was set to seven amino acids per identified peptides and two missed cleavages were allowed. The precursor and fragment ion maximum mass tolerance were set to 10 ppm and 0.02 Da, respectively. Proteins with at least one unique peptide were considered as valid identifications. Deamidation at asparagine was applied as a variable modification to identify N-glycosites of formerly glycosylated peptides and N-linked glycosite-containing peptides were required to contain N-!P-S/T/C motifs. Quantification of SILAC pairs was performed by MaxQuant with standard settings with a minimum ratio count of two. For proteome analysis, variable modifications did not consist of asparagine deamidation and all the other parameters settings were identical with N-glycoproteome analysis.

### Sequence motif analysis

For the de novo derivation of sequence motifs, the Motif-X [61] software was applied to 1525 aligned highly confident glycosylated sites with their surrounding six amino acids to both termini. A minimum occurrence of 15 matches was set for consensus sequence identification. WebLogo [62] as used to create relative frequency plots.

### Gene ontology enrichment analysis

We used Cytoscape [63] and BiNGO [64] for an overview of the cellular component, molecular function, and biological processes. Corresponding Gene Ontology annotations were retrieved from the GOA database [65] (version_2015.12). The hypergeometric model and the Benjamini Hochberg false discovery rate correction were used to calculate statistical significance. A probability value of 0.05 was considered significant.

### Western blot

Proteins were extracted from OVCAR8 and NCI/ADR-RES cells as described above. Equal amounts of protein were separated by SDS-PAGE and electro-transferred to PVDF membranes by semi-dry methods, which were then incubated with primary antibodies after blocking in Tris-buffered saline containing 5% non-fat milk. The immunoreactive protein bands were detected with super-enhanced chemiluminescence substrate after incubating with secondary antibodies. Western blot analyses for all selected proteins were repeated three times and GAPDH was used as loading control.

## SUPPLEMENTARY MATERIALS FIGURES AND TABLES


























