# Supplementary material for: Integrated proteomic and N-glycoproteomic analyses of doxorubicin sensitive and resistant ovarian cancer cells reveal glycoprotein alteration in protein abundance and glycosylation
Source: Oncotarget. 2017 Jan 6;8(8):13413–27. doi: 10.18632/oncotarget.14542 (PMC5355108; doi:10.18632/oncotarget.14542)
Supplement: Supplementary file 1 [file oncotarget-08-13413-s001.pdf]

Integrated proteomic and N-glycoproteomic analyses of doxorubicin sensitive and resistant ovarian cancer cells reveal glycoprotein alteration in protein abundance and glycosylation

Supplementary Materials

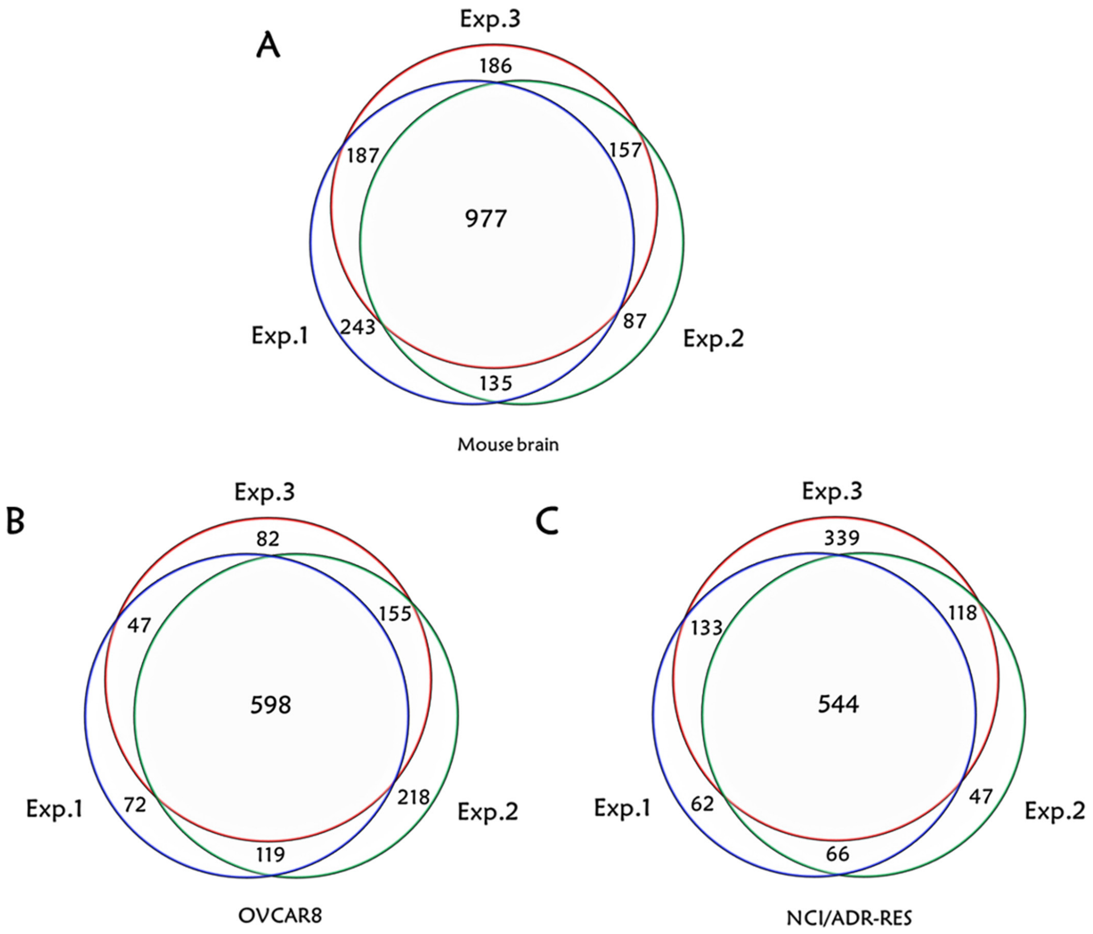

Supplementary Figure 1: Venn diagrams. (**A**) Overlap of the identification of the N-glycosylation sites in mouse brain in triplicates. (**B**) Overlap of the identification of the N-glycosylation sites in OVCAR8 cells. (**C**) Overlap of the identification of the N-glycosylation sites in NCI/ADR-RES cells.

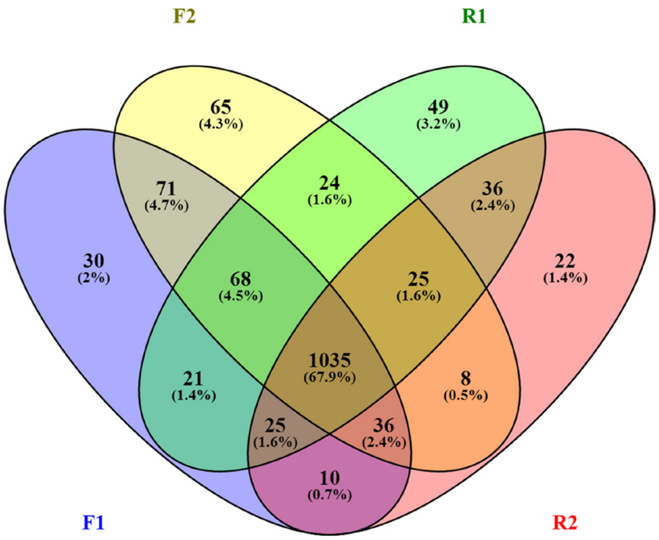

Supplementary Figure 2: A summary of the number of N-glyocsites identified from four independent N-glycoproteome experiments. F1 and F2 results were obtained from two forward SILAC experiments, R1 and R2 results were from two reverse SILAC experiments.

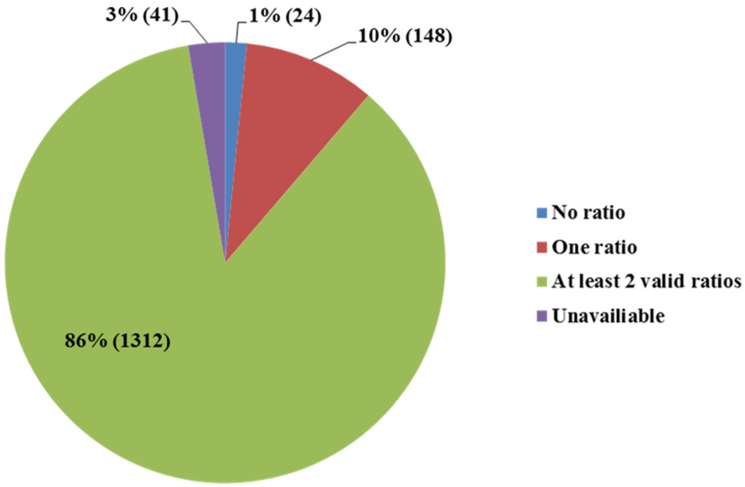

Supplementary Figure 3: Annotation of identified N-glycoproteome (1525) based on the quantitative ratios.

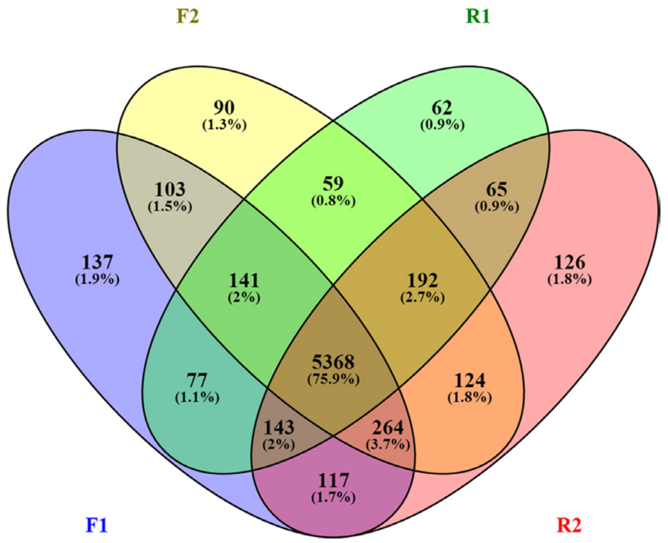

Supplementary Figure 4: A summary of the number of proteins identified from four independent SILAC experiments. F1 and F2 results were obtained from two forward SILAC experiments, R1 and R2 results were from two reverse SILAC experiments.

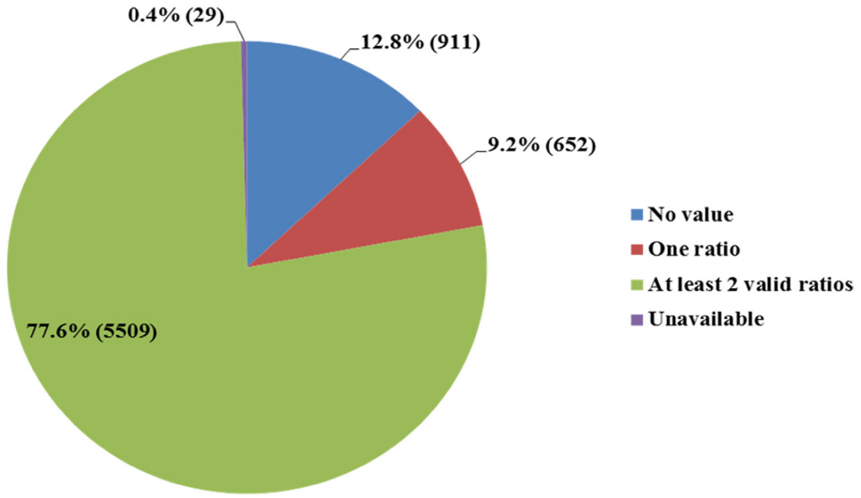

Supplementary Figure 5: Annotation of identified proteome (7101) based on the quantitative ratios.

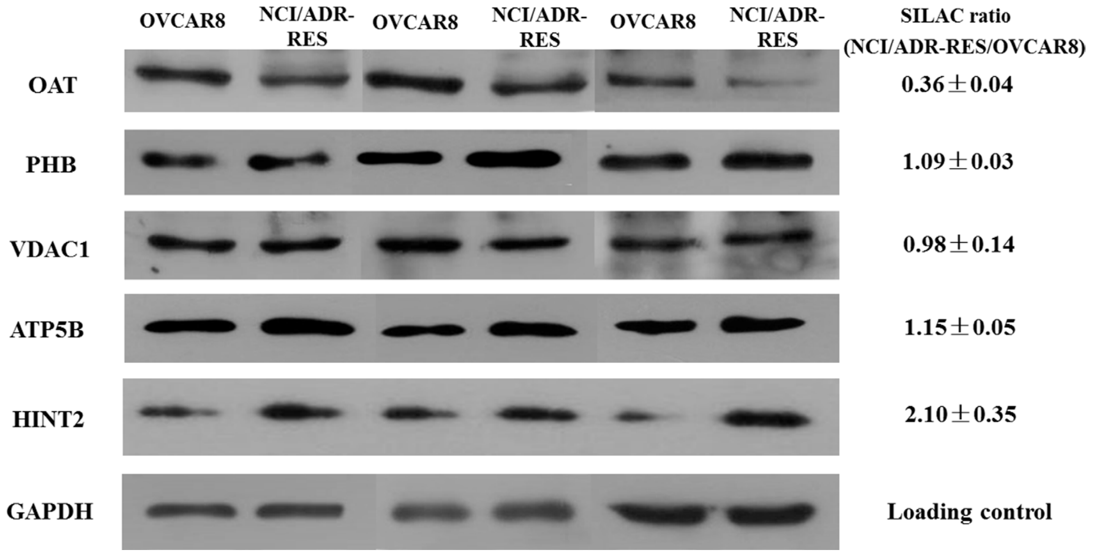

Supplementary Figure 6: Verification of SILAC results by western blotting.

Supplementary Table 1: N-glycosites identified in three replicates in mouse brain. See Supplementary_Table_1

Supplementary Table 2: Data sets of HILIC enrichment specificity in three replicates in mouse brain. See Supplementary_Table_2

Supplementary Table 3: N-glycosites identified in three replicates in OVCAR8 cell and NCI/ADR-RES cells separately. See Supplementary_Table_3

Supplementary Table 4: Data sets of HILIC enrichment specificity in three replicates in OVCAR8 cell and NCI/ADR-RES cells separately. See Supplementary_Table_4

Supplementary Table 5A: N-glycosites identified in the N-glycoproteome of OVCAR8 and NCI/ADR-RES cells. See Supplementary_Table_5A

Supplementary Table 5B: High confidence N-glycosites identified in the N-glycoproteome of OVCAR8 and NCI/ADR-RES cells. See Supplementary_Table_5B

Supplementary Table 5C: High confidence N-glycosites quantified with at least two valid ratios in the N-glycoproteome of OVCAR8 and NCI/ADR-RES cells. See Supplementary_Table_5C

Supplementary Table 6: Potential false postive N-linked glycosylation sites identified by direct analysis of enriched glycopeptides by LC-MS/MS without PNGase F treatment. See Supplementary_Table_6

Supplementary Table 7A: Gene Ontology Analysis, data of an initial overview of the GO distribution provided by GOSlim Generic assignment. See Supplementary_Table_7A

Supplementary Table 7B: Gene Ontology Analysis, data of cellular components that are significantly overrepresented in the N-glycoproteome compared to the entire human proteome. See Supplementary_Table_7B

Supplementary Table 7C: Gene Ontology Analysis, data of molecular functions that are significantly overrepresented in the N-glycoproteome compared to the entire human proteome. See Supplementary_Table_7C

Supplementary Table 7D: Gene Ontology Analysis, data of biological processes that are significantly overrepresented in the N-glycoproteome compared to the entire human proteome. See Supplementary_Table_7D

Supplementary Table 8: Annotation of 253 significantly changed N-glycosite-containing peptides matching to UniProtKB database. See Supplementary_Table_8

Supplementary Table 9: Functional annotation of glycoproteins with significantly changed N-glycosite-containing peptides. See Supplementary_Table_9

Supplementary Table 10A: Protein groups with at least one unique peptide in per protein identified in the comparative proteome of OVCAR8 and NCI/ADR-RES cells. See Supplementary_Table_10A

Supplementary Table 10B: Protein groups quantified with at least two valid ratios in the comparative proteome of OVCAR8 and NCI/ADR-RES cells. See Supplementary_Table_10B

Supplementary Table 11A: Protein annotated as glycoproteins using Uniprot keywords in the comparative proteome of OVCAR8 and NCI/ADR-RES cells. See Supplementary_Table_11A

Supplementary Table 11B: Protein involved in glycan biosynthesis identified in the comparative proteome of OVCAR8 and NCI/ADR-RES cells. See Supplementary_Table_11B

Supplementary Table 12: 570 N-glycosite-containing peptides quantified and calibrated by corresponding protein ratio in SILAC labeling (NCI/ADR-RES:OVCAR8) experiment. See Supplementary_Table_12
